# Inverse pattern of photoreceptor abnormalities in retinitis pigmentosa and cone–rod dystrophy

**DOI:** 10.1007/s10633-012-9348-8

**Published:** 2012-08-05

**Authors:** Midori Yokochi, Danjie Li, Masayuki Horiguchi, Shoji Kishi

**Affiliations:** 1 Department of Ophthalmology, Gunma University School of Medicine, 3-39-22 Showamachi, Maebashi, 371-8511 Japan; 2 Department of Ophthalmology, Fujita Health University School of Medicine, Aichi, Japan

**Keywords:** Cone–rod dystrophy, Full-field electroretinogram, Goldmann perimetry, Optical coherence tomography, Photoreceptor inner and outer segment (IS/OS), Retinitis pigmentosa

## Abstract

**Purpose:**

To determine the characteristics of the photoreceptor abnormalities in retinitis pigmentosa (RP) and cone–rod dystrophy (CRD).

**Methods:**

We evaluated the photoreceptor abnormalities using spectral-domain optical coherence tomography (SD-OCT) in 28 patients with RP and 17 patients with CRD. The OCT images and full-field electroretinograms were obtained from 21 eyes in normal subjects who were age-matched to patients with RP and CRD and served as controls.

**Results:**

Eyes with RP and CRD had markedly decreased rod responses (6.5 and 57.5 % of normal value), maximal responses (9.6 and 51.6 %), cone (16.5 and 25.8 %), and 30-Hz flicker responses (17.8 and 30.1 % of normal value), and their *P* values were smaller than 0.0003. On comparison of ERG data between RP and CRD, they had statistically significant differences in rod responses (*P* < 0.0003) and maximal responses (*P* < 0.0003). However, there were no statistical differences in cone response and a weak difference in 30-Hz flicker responses (*P* < 0.017). The best-corrected visual acuity was −0.03 ± 0.09 (logMAR, mean ± standard deviation [SD]) in eyes with RP, but 0.57 ± 0.54 in eyes with CRD. SD-OCT showed that eyes with RP had an intact reflective line at the junction between the photoreceptor inner and outer segment (IS/OS) at the fovea, while eyes with CRD had no IS/OS. The extent of the central visual field was correlated with the IS/OS length at the macula in eyes with RP.

**Conclusion:**

The distribution patterns of the IS/OS line help to differentiate between RP and CRD.

## Introduction

Retinitis pigmentosa (RP) is an inherited rod–cone dystrophy characterized by night blindness, photophobia, and loss of the peripheral visual field. Although the fundus appears normal in the early stage of the disease, attenuated retinal vessels, intraretinal bone spicule pigmentation in the midperipheral fundus, and a waxy pallor of the optic disk develop in the advanced stage.

Full-field electroretinography (ERG) is essential for diagnosing RP, which is characterized by markedly reduced or no rod response, and evaluating the disease severity. The cone response is absent or occasionally recordable in the relatively early stage of the disease.

Cone–rod dystrophy (CRD) is characterized by early loss of visual acuity (VA) and color vision, with subsequent progressive peripheral visual field loss [1–3]. In CRD, the cone function deteriorates first, and rod dysfunction develops later. Differentiating between pure cone dystrophy and CRD is difficult, because some degree of rod dysfunction develops as the cone dystrophy progresses. Thus, the definitions that have been used conventionally are “cone dystrophy for retinal diseases with predominant cone dysfunction with later onset and mild rod involvement,” and “CRD for retinal dystrophies with early onset cone dysfunction followed shortly thereafter by significant rod disease” [1]. In both rod–cone (RP) and CRDs, the symptoms occasionally overlap, making the differential diagnosis difficult.

Optical coherence tomography (OCT) is a powerful tool for diagnosing retinal diseases [4]. Spectral-domain OCT (SD-OCT), with 5-μm axial resolution, detects the reflective line at the junction between the photoreceptor inner and outer segment (IS/OS) and the external limiting membrane (ELM). SD-OCT can identify topographic variations in photoreceptor damage by evaluating the IS/OS, ELM, and retinal thickness [5]. Using SD-OCT, we studied the characteristics of RP and CRD.

## Methods

This study included 28 eyes of 28 patients (15 men, 13 women) with RP and 17 eyes of 17 patients (10 men, 7 women) with CRD who were examined at Gunma University Hospital between November 2007 and February 2012.

No eyes had any other ocular diseases. All eyes were phakic, and the patients had no history of ocular surgery. RP and CRD were diagnosed based on subjective symptoms, ERG, color fundus photographs, and Goldmann perimetry. Patients with cystoid macular edema, which affects the central foveal thickness, those with restricted cone dystrophy, and those with advanced disease with VA below hand motion and nonrecordable Goldmann visual fields were excluded. If ERG was properly performed, all the patients were included regardless of a poor electrical response. We adopted the data for this study from the examinations at which we first obtained the best-corrected VA (BCVA), OCT, ERG, and Goldmann perimetry data at the same time.

For a statistical analysis, we chose only one eye from each patient. We selected the eye with better BCVA as a sample. If both eyes had same BCVA, we chose the right eye.

For normal controls with good visual acuity (all BCVAs were more than −0.08 logMAR), we measured the central foveal thickness in 21 right eyes of 21 subjects (age range, 13–79 years; mean, 53.8 ± 18.6 years) and evaluated the ERG recordings in the 21 normal right eyes.

### VA and visual fields

The BCVA was measured as the decimal VA using a Landolt chart and converted to the logarithm of the minimum angle of resolution (logMAR) for statistical analysis. We examined the Goldmann perimetry results in 45 eyes of 45 patients. The eyes with RP were classified into three types of visual field defects: type A with a central visual field of 5° or less, type B with a central visual field of 5–10°, and type C with more than 10°.

### OCT

We performed SD-OCT (Cirrus high-definition OCT, Carl Zeiss Meditec, Inc., Dublin, CA) in all 45 eyes with ocular diseases and in 21 normal eyes and obtained cross-sectional images of 6-mm horizontal and vertical scans through the central fovea. We measured the central foveal thickness (CFT), defined as the distance between the inner surface of the central fovea and the outer border of the retinal pigment epithelium (RPE). We assessed the retinal microstructure, particularly the integrity of the IS/OS. In cases with IS/OS at the fovea, we measured the length in the horizontal and vertical scans and obtained an average value.

### ERG

Full-field ERGs were recorded after pupillary dilation with 1 % tropicamide and 2.5 % phenylephrine hydrochloride using a light-emitting diode–built-in contact lens electrode (Mayo Nagoya Japan) [6–8]. The reference and ground electrodes were attached to the patients’ forehead and earlobe, respectively. The signals were amplified with a bandpass between 0.3 and 300 Hz. The rod ERG was elicited by white stimuli with a luminance of 2.49 log cd/m^2^ with a 30-μ s duration after 20 min of dark adaptation, and then the maximal ERG was elicited by white stimuli with a luminance of 3.0 log cd/m^2^ and a 3-ms stimulus duration. The cone ERGs were also elicited by white stimuli with a luminance of 3.0 log cd/m^2^ and a 3-ms duration, and the 30-Hz flicker ERGs were elicited with white stimuli with a peak luminance of 2.0 log cd/m^2^ after 10 min of light adaptation. The cone and 30-Hz flicker ERGs were recorded on white 1.39 log cd/m^2^ background illumination. These stimulus conditions conformed as much as possible to the ERG standards recommended by the International Society for Clinical Electrophysiology of Vision (ISCEV) [9].

To diagnose RP and CRD, we measured the amplitudes of the rod b-wave, maximal b-wave, cone b-wave, and 30-Hz flicker b-wave.

### Statistical analyses

Statistical analysis was performed with commercial software (Excel for Windows; 4 steps Excel statistics-2nd). In general, associations between two numerical variables of independence were examined by using Spearman’s rank correlation and the Mann–Whitney *U* test. The criterion significance was assessed at the *P* < 0.05 level. To compare three groups, a Bonferroni correction was used to adjust for multiple pair-wise comparisons; then, criterion significance was assessed at the *P* < 0.017 level (*n* = 3). A simple linear regression analysis was performed to identify a correlation between the length of the IS/OS at the fovea and the ERG data (including four amplitudes of rod, maximal, cone, and 30-Hz flicker responses).

The research was conducted according to the institutional guidelines of Gunma University and conformed to the tenets of the Declaration of Helsinki. The patients provided informed consent after they received a full explanation of the study procedures.

## Results

The age range of the 21 normal control subjects was 13–79 years (mean ± SD: 53.8 ± 18.6 years), that of RP patients was 8–72 years (47.2 ± 17.5), and that of CRD patients was 5–85 years (52.2 ± 17.8). The age distributions showed no significant differences in the three groups (RP vs normal control: *P* = 0.18; RP vs CRD: *P* = 0.41; CRD vs normal control: *P* = 0.63; Mann–Whitney *U* test).

The BCVAs were −0.03 ± 0.09 (logMAR, mean ± standard deviation [SD]) (range, 0.22 to −0.18 logMAR) in 28 eyes with RP and 0.57 ± 0.54 (logMAR, mean ± SD) (range, 2.00 to −0.08 logMAR) in 17 eyes with CRD. The BCVA in CRD patients was lower than in RP patients (*P* < 0.0003, multiple comparison test of Bonferroni, *n* = 3) and normal controls (*P* < 0.0003). The BCVA in RP patients had not significantly decreased (*P* > 0.017) compared with normal controls.

**Fig. 1:**
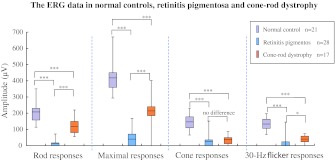
Amplitudes of rod, maximal, cone, and 30-Hz flicker responses in normal controls, in patients with retinitis pigmentosa (*RP*), and in patients with cone–rod dystrophy (*CRD*). The *boxes* represent the 25–75 % interquartile ranges. The horizontal lines represent mean values, and the bars represent 5–95 % confidence intervals. All responses of RP and CRD were significantly lower compared with normal controls. On comparison between RP and CRD patients, there were significant differences in rod and maximal responses, but there was no difference in cone (*P* = 0.035) and weak in 30-Hz flicker (*P* = 0.008) responses. The criterion significance was assessed at the *P < 0.017; **P < 0.003; ***P < 0.0003. (Mann–Whitney *U* test was corrected for multiple comparisons by the Bonferroni method.)

The ERG data and their statistical analysis in normal controls and RP and CRD patients are summarized in Fig. 1. We used the statistical method of Mann–Whitney *U* test with criterion significance levels between two groups of *P < 0.05, **P < 0.01, and ***P < 0.001. The asterisks indicate significant differences. Because of the comparison between three groups in this study, we corrected significant difference level to *P < 0.017, **P < 0.003, and *** *P* < 0.0003 (*n* = 3) using the Bonferroni method. For ERGs obtained from 28 eyes with RP, the amplitudes of the rod b-wave and maximal b-wave were 13.4 ± 24.6 (mean ± SD) and 40.2 ± 53.9 μV, respectively. Because the means of rod and maximal b-wave of 21 normal eyes were 207.9 ± 63.7 and 418.2 ± 97 μV, the responses of RP patients were only 6.5 and 9.6 % of normal controls. The amplitudes of the cone b-wave and 30-Hz flicker in RP patients were 24.2 ± 34 and 23.7 ± 37.9 μV, respectively, which were 16.5 and 17.8 % of normal controls (146.6 ± 43.1 and 133.3 ± 38 μV, respectively). There were statistically significant strong differences between RP patients and normal controls in rod (*P* < 0.0003), maximal (*P* < 0.0003), cone (*P* < 0.0003), and 30-Hz flicker responses (*P* < 0.0003).

In the 17 eyes with CRD, the means of the rod, maximal, cone, and 30-Hz flicker b-wave were 119.5 ± 44.7, 216 ± 84.8, 37.8 ± 27.1, and 40.2 ± 24.1 μV, respectively, which were 57.5, 51.6, 25.8, and 30.1 % of normal controls, respectively. There were statistically significant strong differences between CRD patients and normal controls in rod (*P* < 0.0003), maximal (*P* < 0.0003), cone (*P* < 0.0003), and 30-Hz flicker responses (*P* < 0.0003).

On comparison of ERG data between RP and CRD patients, they had statistically significant differences in rod responses (*P* < 0.0003) and maximal responses (*P* < 0.0003). However, there was no statistical differences in cone b-wave response (*P* = 0.04) and weak difference in 30-Hz flicker responses (*P* < 0.017).

**Fig. 2:**
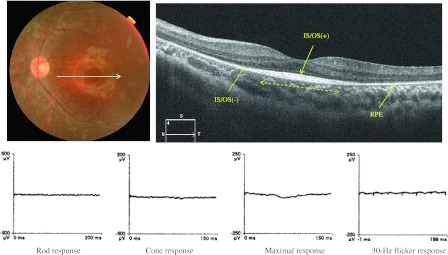
A 13-year-old boy with RP. His BCVA was 0.10 (logMAR) in left eye. The posterior fundus had a normal color, but the midperiphery was atrophic. OCT shows an intact IS/OS in the macular area (*dashed line*) but no IS/OS outside the macula in the 6-mm horizontal scan. The rod, maximal, cone, and 30-Hz flicker responses were nonrecordable bilaterally

**Fig. 3:**
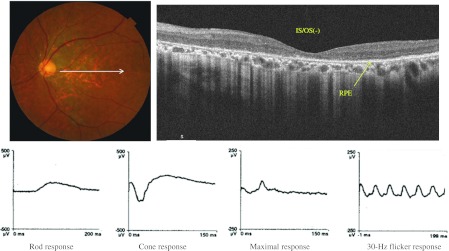
A 58-year-old man with CRD. The BCVA was 1.00 (logMAR) in the left eye. The fundus showed diffuse atrophy in the posterior pole. OCT showed no IS/OS and an attenuated choroid (*arrow*) in the posterior fundus. All responses in the ERG were decreased, the rod b-wave was 115.6 μV, the maximal b-wave was 267 μV, the cone b-wave was 60.3 μV, and the 30-Hz flicker response was 55.6 μV

For SD-OCT, 21 normal eyes had an intact IS/OS line throughout the horizontal and vertical sections. The IS/OS was present at the fovea, but absent outside of the macula in 28 eyes with RP. In the 6-mm horizontal sections, the length of the intact IS/OS line varied in the eyes with RP (Fig. 2). In 17 eyes with CRD, the IS/OS line was not detected along with atrophy of the RPE throughout the 6-mm section (Fig. 3).

### Length of the IS/OS line in RP

**Fig. 4:**
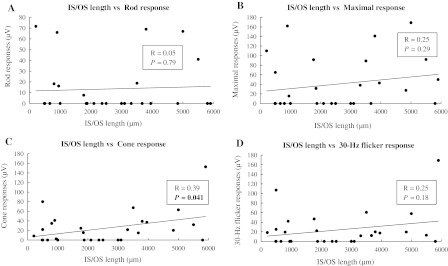
The ERG responses and the length of the junction between the IS/OS in eyes with RP. The cone response was significantly correlated with the length of IS/OS line at the fovea (**c**). The correlation coefficient (R) was 0.39, and *P* value was 0.041 (simple linear regression analysis). However, the rod, maximal, and 30-Hz flicker responses were not significantly correlated with the length of IS/OS line (**a**, **b** and **d**); their correlation coefficients (Rs) were 0.05, 0.2, and 0.25; and *P* values were larger than 0.05

**Fig. 5:**
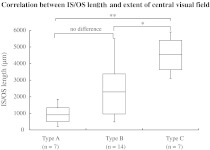
The *box* plot indicates the IS/OS length of eyes with retinitis pigmentosa and the extent of central visual field which were classified *type* *A* (visual field of 5° or less), *B* (5–10°), and *C* (exceeding 10°). The *boxes* represent the 25–75 % interquartile ranges. The *horizontal lines* represent mean values, and the *bars* represent 5–95 %. There were significant differences between types B and C (*P = 0.0045, Mann–Whitney *U* test) and A and C (**P = 0.0017), and no significant differences between *A* and *B* (*P* = 0.021). The criterion significance level of *P* < 0.05 was corrected for multiple comparisons by the Bonferroni method, to *P < 0.017, **P < 0.003

There was no statistically significant correlation (Spearman’s rank correlation coefficient: *r* = 0.07, *P* = 0.72) between the BCVA and the length of the IS/OS line at the fovea in the 28 eyes with RP. For the ERG, the cone responses were significantly greater in eyes with a longer IS/OS line (Fig. 4c). The correlation coefficient (*R*) was 0.39, *P* = 0.041 (simple linear regression analysis). However, there was no statistically significant correlation between the length of the IS/OS line and the rod (*R* = 0.05, *P* = 0.79; Fig 4a), maximal (*R* = 0.25, *P* = 0.29; Fig. 4b), and 30-Hz flicker (*R* = 0.25, *P* = 0.18; Fig 4d) responses. The central visual field was wider in eyes with a longer IS/OS line (Fig. 5). The length of the IS/OS line in seven eyes with a central visual field of 5° or less (type A) was 912.7 ± 646.7 μm (mean ± standard deviation [SD]), 2,289.9 ± 1490.1 μm (mean ± SD) in 14 eyes with a central visual field of 5–10° (type B), and 4,557.4 ± 1129.1 μm in seven eyes with a central visual field exceeding 10° (type C). There were statistically significant correlations between types B and C (*P = 0.0045, Mann–Whitney *U* test) and A and C (**P = 0.0017) and no statistically significant correlation between A and B (*P* = 0.021). The criterion significance levels of *P < 0.05 and **P < 0.01 were corrected for multiple comparisons by the Bonferroni method to *P < 0.017 and **P < 0.003.

### Central foveal thicknesses

**Fig. 6:**
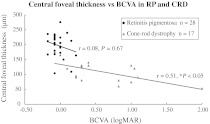
Between central foveal thickness and the best-corrected visual acuity (BCVA), there was a moderate correlation (*gray line*) in eyes with cone–rod dystrophy (CRD) (Spearman’s rank correlation coefficient: *r* = 0.51, *P* = 0.04.), but it was not significantly correlated (*black line*) with retinitis pigmentosa (RP) (*r* = 0.08, *P* = 0.67)

The CFTs of normal eyes ranged from 180 to 250 μm (mean ± standard deviation: 218 ± 20). The mean CFT was 198 ± 38 μm in the 28 eyes with RP and 109 ± 35 μm in the 17 eyes with CRD. There was no significant difference in the CFT between RP and normal eyes (*P* = 0.04 Mann–Whitney *U* test corrected by the Bonferroni method), but the CFT in eyes with CRD was markedly thinner (*P* < 0.0003) than in normal controls and eyes with RP. A graph of the CFTs and BCVA (Fig. 6) shows that the eyes with RP had good BCVA, and the CFT (198 ± 38 μm) was slightly thinner (the mean CFT in normal eyes: 218 μm), but thicker than in eyes with CRD. No correlation (Spearman’s rank correlation coefficient: *r* = 0.08, *P* = 0.67) was seen between the CFT and BCVA. Eyes with CRD were widely distributed on the graph, but there was a significant positive correlation (*r* = 0.51, *P* = 0.04) between the CFT and BCVA. A central scotoma was seen in 17 eyes with CRD, but not in 28 RP eyes with an IS/OS. The 28 eyes had a concentric visual field defect.

## Discussion

In the current study, the ERG showed severely decreased rod responses and markedly reduced cone responses in the 28 eyes with RP compared with average values from eyes of normal controls. The 17 eyes with CRD also had a significantly decreased cone response, but a relatively preserved rod response.

SD-OCT showed that the distribution pattern of the IS/OS line in RP was inverse to that of CRD, which was diagnosed by conventional methods including ERG, Goldmann perimetry, fundus photography, and VA measurement. In RP, the IS/OS line was present at the fovea with varying lengths and absent outside the macula; in CRD, the IS/OS line was absent at the fovea (Fig. 2, 3). In RP, the cone responses were positively correlated with the length of the IS/OS line at the macula; however, the length of the IS/OS was not correlated with the rod, maximal, and 30-Hz flicker responses (Fig. 4). Our results showed that the length of the IS/OS correlated with the sizes of central visual field in RP (Fig. 5). Using SD-OCT and Goldmann perimetry, Fischer et al. [10] reported that eyes with a longer IS/OS at the fovea had a larger visual field.

The CFT was relatively good in RP eyes with foveal IS/OS, but markedly thinner in CRD eyes with no IS/OS. In patients with RP with an intact IS/OS at the fovea, the CFT was comparable to that in normal subjects, while it was significantly thinner in CRD with no IS/OS at the fovea. Although no IS/OS was detected at the fovea in CRD, the BCVA was correlated with the CFT in CRD, suggesting that there were residual photoreceptors at the fovea even in CRD.

The primary lesion in RP and CRD is seen on OCT in the photoreceptor outer segments. The IS/OS line represents hyperreflectivity at the junction between the IS/OS, because the structure of the outer segment simulates multilaminated disks where the OCT measurement beam generates high reflectivity (seen as a highly reflective IS/OS line). Thus, the integrity of the IS/OS line reflects that of the photoreceptor outer segment. It is noteworthy that the characteristic photoreceptor abnormalities in RP and CRD are well reflected in a 6-mm OCT image through the fovea despite the wide distribution of the rods and cones in the entire fundus. In human eyes, rods account for 95 % of all photoreceptors; cones comprise only 5 % of the photoreceptors. The cone density peaks acutely at the fovea, but decreases in the perifoveal area. Although rods are absent at the fovea within a 350-μm diameter, the rod density increases sharply outside the fovea and peaks in the annular zone about 20° outside the fovea. Since a 6-mm horizontal scan through the fovea encompasses the posterior pole from near the optic disk margin to 3 mm temporal to the fovea, it involves the peak of the cone distribution curve and the ascending slope of the rod distribution close to the peak [11, 12]. Because of the high density of rods and cones, the 6-mm macular area is the optimal site that reflects the morphologic changes in the rods and cones.

In the current study, the CFT and BCVA were correlated positively in CRD but not in RP (Fig. 6). Using ultrahigh-resolution OCT, Witkin et al. [13] reported that the CFTs did not differ significantly between normal individuals and patients with RP (rod–cone dystrophy), which also was seen in the present study. In contrast to our results, Fischer et al. [10], Aizawa et al. [14], Witkin et al. [13], and Sandberg et al. [15] reported a correlation between CFT and BCVA in RP. This contradiction appears to be due to the fact that the current patients with RP still had relatively good VA and patients with advanced RP were excluded. In CRD, the cone outer segment disappears first, and then the outer nuclear layer is attenuated. In the current study, although CRD eyes had no IS/OS line at the fovea, their visual acuity was correlated with the CFT. This suggested that some cone outer segment or photoreceptor cells still survived in eyes with a thicker CFT.

Oishi et al. [16] studied the electrical responses and the integrity of the IS/OS line in the macular area using focal ERGs with a 15-degree stimulus and SD-OCT. The focal electrical responses were well correlated with the length of the intact IS/OS. The current study also found this correlation in the cone response using ERG (Fig. 6). The absence of or decreased length of the IS/OS line was correlated with the macular function. Sugita et al. [11] reported that the length of the intact IS/OS and macular volume measured by SD-OCT were correlated weakly with the amplitude of the focal macular ERG.

In conclusion, the inverse pattern of an intact IS/OS line in the macular area corresponded well with the different ERG patterns between RP and CRD. The length of the IS/OS line at the fovea predicted the BCVA and central visual field in patients with RP. Although eyes with CRD had no IS/OS line at the fovea, their BCVA was moderately correlated with the CFT.
